# Adverse childhood experiences and future self-rated health: a prospective cohort study

**DOI:** 10.1186/s12889-021-10941-3

**Published:** 2021-05-12

**Authors:** Alexander Jahn, Timmi K. Rysgaard, Johan Hviid Andersen, Trine Nøhr Winding

**Affiliations:** Department of Occupational Medicine, Danish Ramazzini Centre, University Research Clinic, Regional Hospital West Jutland, Gl. Landevej 61, 7400 Herning, Denmark

**Keywords:** Adverse childhood experiences, Self-rated health, Logistic regression

## Abstract

**Background:**

Negative life events (re) occurring during childhood is often described as adverse childhood experiences (ACEs) and may have long-lasting negative effects on health. Previous studies on the association between ACEs and self-rated health (SRH) have primarily been focusing on chronic diseases in elderly, non-Scandinavian populations using a cross-sectional design. The aim of the study was to examine the associations between ACEs and SRH in early adulthood and to investigate if disadvantageous health-behavioral strategies explain the association between ACEs and SRH.

**Methods:**

A prospective cohort study using data from The West Jutland Cohort Study (*N* = 2.255). Baseline data on exposure to ACEs were collected from surveys at the age of 15 and 18 and respondents were categorized into having experienced 0, 1–2, 3 or > 4 ACEs. The outcome SRH stems from surveys at the age of 21 and 28 and was dichotomized into moderate and good SRH. The association between ACE-categories and SRH at age 21 and 28 were analyzed separately by logistic regression with a two-step adjustment model, adjusting for potential confounders and disadvantageous health-behavioral strategies.

**Results:**

More than half of the participants reported at least one ACE (56.3%) with “*bullying*” and “*loss of parent, parental separation or divorce*” being the most prevalent. Participants who reported > 4 ACEs, compared to those with 0 ACEs, had a 2.6-fold increased odds (95% CI 1.3; 5.1) of having moderate SRH at the age of 21, and a 2.7-fold increased odds (95% CI 1.4; 5.4) of moderate SRH at the age of 28 years, when adjusted for potential confounders. Further, small attenuations of the estimates were seen when adjusting for disadvantageous health-behavioral strategies. A significant exposure response relationship between the ACE-categories and moderate SRH were seen both at age 21 and 28.

**Conclusion:**

The study showed an association between ACEs and moderate SRH in young adulthood, and experiencing multiple ACEs increased the odds of reporting moderate SRH. Information on ACEs could help identifying people with a higher risk of future health problems and accentuates a growing need for early prevention in homes with children who has experienced adverse events.

## Background

Self-rated Health (SRH) can play an important role in investigating potential early health problems due to its strong predictive ability of future morbidity and mortality even after adjustments for risk factors known to influence SRH and mortality [1–3]. Rating one’s own health as poor compared to excellent has been associated with a two-fold increased mortality risk in several reviews [1, 3].

It is well documented that experiencing adversities in childhood is common [4, 5] and in a recent review Hughes et al. found that more than 57% of 252.467 participants in 37 studies reported at least one adversity in their childhood [6]. Adverse childhood experiences (ACEs) cover a variety of (re) occurring events spanning from household dysfunctionalities, alcohol abuse, violence- and bullying to sexual abuse and mental illness of a parent [4]. In Denmark, in 2020, it is estimated that one out of six children have experienced physical violence in the household and 5% of all children in Denmark have a parent that have a high alcohol consumption [7]. In 2019, the Local Government Denmark, which is an interest organization of the 98 Danish municipalities, estimated the net operating expenses for all municipalities to be approximately 15.7 billion Danish kroner (DKK) regarding socially vulnerable children and adolescents who received preventive safety precaution [8].

A child’s development during early childhood builds the foundation for learning, relationships, problem solving etc. and is therefore a highly sensitive period. Thus, experiencing childhood adversities can have a negative long-lasting profound impact on a child’s future development and health [9–11].

The first major study of the associations between ACEs and adult health was conducted in 1998 by Felitti et al. [4]. Commonly known as the ACE study, it showed strong relationships between the leading causes of death in the US and experiencing ACEs in the childhood. Afterwards, a global network, including World Health Organization (WHO) and the United States Centers for Disease Control and Prevention, was established focusing on the long-term consequences of ACEs [12]. This resulted in the development of the Adverse Childhood Experiences International Questionnaire (ACE-IQ) [13] to be integrated in health surveys covering in total 13 different ACE-categories covered by 29 questions.

ACEs have been associated with different health problems like chronic diseases, mental illness and functional limitations. Simultaneously, multiple ACEs have showed strong exposure response relationships with increasing risk of these adverse health problems [4, 6, 11, 14, 15]. In a societal perspective, individuals experiencing ACEs are more likely to be unemployed and have increased health care utilization [16, 17]. Moreover, ACEs have been found to cause disadvantageous health-behavioral strategies in adulthood leading to smoking, higher alcohol consumption, drug abuse and risky sexual behavior [4, 18–20]. It is evidentially outlined that poor mental well-being and low socio-economic status (SES), including low parental education and income, are potential risk factors for reporting ACEs [5, 14, 21–28].

Since most previous studies on the association between ACEs and SRH are from the US, a knowledge gap exists from countries driven by different models of social security and healthcare systems. It is possible that ACEs affect health differently in countries with a more prominent welfare system, e.g. Denmark. To our knowledge, no previous studies investigating the association between ACEs and SRH in Denmark have been conducted and only two studies from Scandinavia were found [21, 26]. Overall, the current evidence on the association between ACEs and SRH are dominated by cross-sectional studies studying elderly populations using a retrospective approach to collect information on ACEs, and the majority of these studies suffer from a risk of both survival- and information bias.

Amemiya et al. [21] conducted a cross-sectional study comparing older adults in Japan (+ 69 years) with older adults in Finland (+ 64 years). The study used retrospective collection of information, including only three types of ACEs. Kestilä et al. [26] also conducted a cross-sectional study consisting of young Finnish adults (age 18–39). The study included 11 types of ACEs using retrospective self-reported collection of information and analyzed each ACE separately without using categories (multiple ACEs). Therefore, to understand how ACEs affect SRH in a Danish population, a prospective cohort study focusing on early signs of poor self-rated health in adult life is needed.

The primary aim of this study was to examine the association between ACEs and SRH in early adulthood in a young Danish population and to investigate the impact of multiple ACEs on SRH. Secondly, the study aimed to investigate if disadvantageous health-behavioral strategies explain the association between ACEs and SRH.

## Methods

### Design and population

This prospective cohort study used data from the ongoing West Jutland Cohort Study which investigates different aspects of social inequalities and health. It is a complete regional cohort of 3.681 young people born in 1989 and still living in the county of Ringkoebing, Denmark, in early April 2004. The recruitment took place at elementary schools within the county where the baseline questionnaire was filled out during school hours. To include those not in schools on the day of data collection, the questionnaire was sent by post to the home address. In total, 3.054 young people responded in 2004 (age 14/15), resulting in an initial response rate of 83%. So far, The West Jutland Cohort Study consists of three follow-up rounds collected in 2007 (age 17/18), 2010 (age 20/21) and 2017 (age 27/28, [29]). The follow-up questionnaires were distributed to the complete source population at each follow-up round except to those who did not want to participate, had left the country or died – regardless of prior response.

**Fig. 1:**
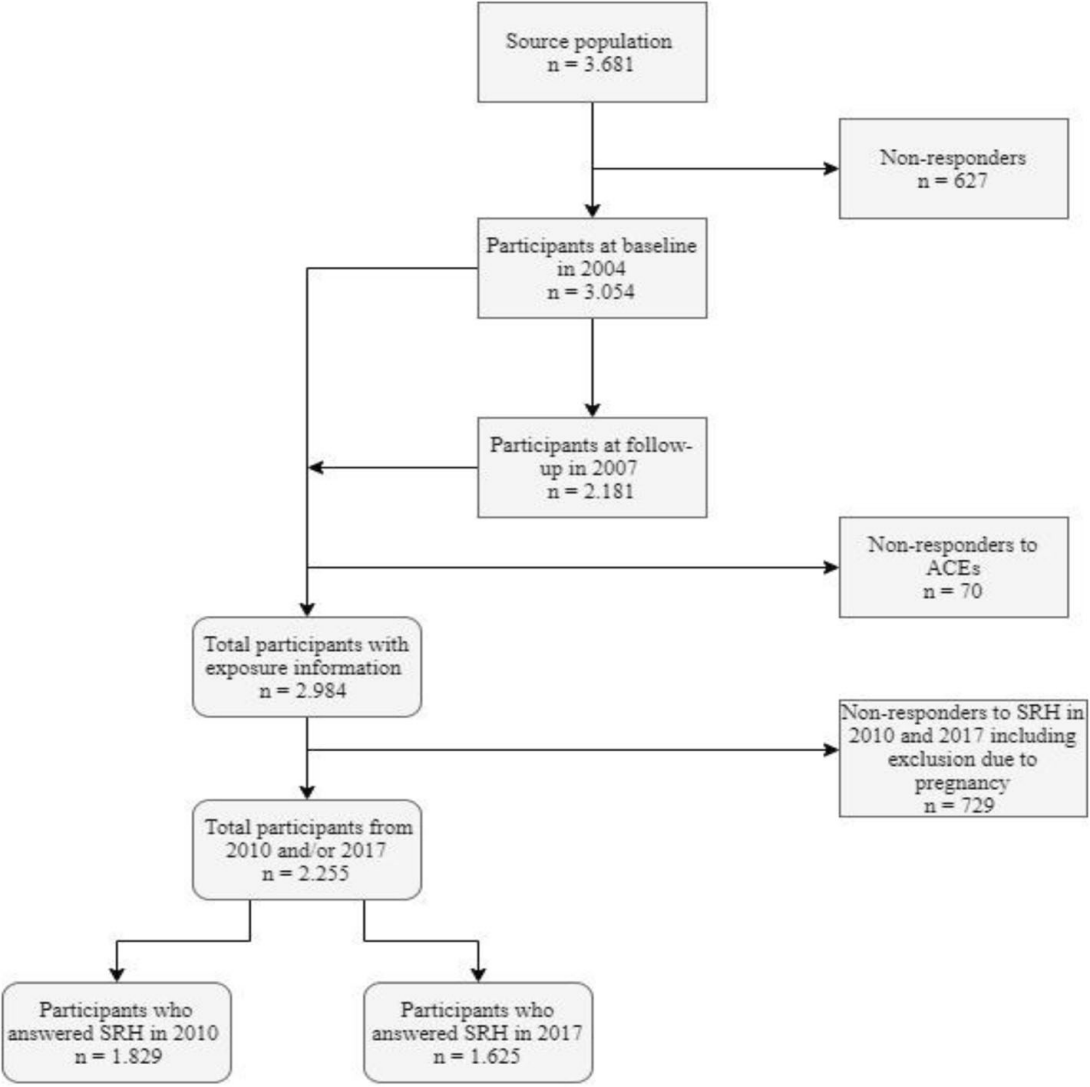
Distribution of participants, non-responders and excluded participants in 2004, 2007, 2010 and 2017

Participants in this study included those who had answered all questions regarding ACEs in 2004 and questions regarding SRH in 2010 and/or 2017, resulting in 2.255 participants for the analysis. The exclusions regarding attrition and missing data are displayed in Fig. 1.

### Outcome

The primary outcome measure was SRH. It was derived from questionnaires in 2010 and 2017 and assessed by the single item question “in general, would you say your health is …” with the response categories “excellent”, “very good”, “good”, “less good” and “poor”. These categories were dichotomized into “good” (excellent/very good) and “moderate” (good/less good/poor) SRH.

### Exposure

Information about ACEs were collected at age 15 and 18 by using single items and abbreviated, validated scales from The West Jutland Cohort Study. We generated proxy variables from both questionnaires at age 15 and 18 in order to be consistent with the WHO’s ACE-IQ binary version [13] and hereby complied with the age criteria of 18 for adversity experiences in the childhood given by WHO. The construction of the following six ACE-categories: 1. Abuse, 2. Alcohol and/or drug abuser in the household, 3. Loss of parent, separation or divorce, 4. Emotional neglect, 5. Bullying and 6. Witnessing violence was inspired by ACE-IQ binary version comparing questions and their topics as seen in Table 1 and explained in details below. Participants were defined as exposed to an ACE-category if they responded “yes” to one or more questions in the respective category.

**Table 1:** Construction of Adverse Childhood Experiences

ACE-categories from WHO’s ACE-IQ	Questions from the ACE-IQ	Questions from The West Jutland Cohort Study questionnaire	Proxy variables
Physical abuse	Did a parent, guardian or other household member spank, slap, kick, punch or beat you up? Did a parent, guardian or other household member hit or cut you with an object, such as a stick (or cane), bottle, club, knife, whip etc.?	Have you been abused by someone you knew?^a^	Abuse
Emotional abuse	Did a parent, guardian or other household member yell, scream or swear at you, insult or humiliate you? Did a parent, guardian or other household member threaten to, or abandon you or throw you out of the house?	Have you been abused by someone you knew?^a^	
Contact sexual abuse	Did someone touch or fondle you in a sexual way when you did not want them to? Did someone make you touch their body in a sexual way when you did not want them to? Did someone attempt oral, anal, or vaginal intercourse with you when you did not want them to? Did someone actually have oral, anal, or vaginal intercourse with you when you did not want them to?	Have you been abused by someone you knew?^a^	
Alcohol and/or drug abuser in the household	Did you live with a household member who was a problem drinker or alcoholic, or misused street or prescription drugs?	Have any of your parents abused alcohol or drugs to an extent where it caused problems in the family?^a^	Alcohol and/or drug abuser in the household
One or no parents, parental separation or divorce	Were your parents ever separated or divorced? Did your mother, father or guardian die?	Were your parents ever separated or divorced?^a,b^ Have you lost any of your parents because they died?^a^	Loss of parent, parental separation or divorce
Emotional neglect	Did your parents/guardians understand your problems and worries? Did your parents/guardians really know what you were doing with your free time when you were not at school or work?	He/she seems emotionally attached to me.^a^ He/she understands my worries and problems.^a^	Emotional neglect
Bullying	Were you bullied?	Have you been bullied at school the last 6 months?^a^ Have you been bullied in an unpleasant way at school during the last 6 months?^b^ Have you been bullied in an unpleasant way at work during the last 6 months?^b^	Bullying
Household member treated violently	Did you see or hear a parent or household member in your home being yelled at, screamed at, sworn at, insulted or humiliated? Did you see or hear a parent or household member in your home being slapped, kicked, punched or beaten up? Did you see or hear a parent or household member in your home being hit or cut with an object, such as a stick (or cane), bottle, club, knife, whip etc.?	Have you witnessed a very violent event?^a^	Witness to violence
Community violence	Did you see or hear someone being beaten up in real life? Did you see or hear someone being stabbed or shot in real life? Did you see or hear someone being threatened with a knife or gun in real life?	Have you witnessed a very violent event?^a^	
Collective violence	Were you forced to go and live in another place due to any of these events? Did you experience the deliberate destruction of your home due to any of these events? Were you beaten up by soldiers, police, militia, or gangs? Was a family member or friend killed or beaten up by soldiers, police, militia, or gangs?	Have you witnessed a very violent event?^a^	
Physical neglect	Did your parents/guardians not give you enough food even when they could easily have done so? Were your parents/guardians too drunk or intoxicated by drugs to take care of you? Did your parents/guardians not send you to school even when it was available?	Could not be established with questions from the West Jutland Cohort Study	Could not be included
Incarcerated household member	Did you live with a household member who was ever sent to jail or prison?	Could not be established with questions from the West Jutland Cohort Study	Could not be included
Someone chronically depressed, mentally ill, institutionalized or suicidal	Did you live with a household member who was depressed, mentally ill or suicidal?	Could not be established with questions from the West Jutland Cohort Study	Could not be included

Questions from the ACE-IQ could only be answered with yes/no.

^a^questions asked at age 15. ^b^questions asked at age 18

*Abuse* was measured by the question “Have you been abused by someone you knew?” at age 15 with the response categories “yes” or “no”.

*Alcohol and/or drug abuser in the household* was measured by the question “Have any of your parents abused alcohol or drugs to an extent where it caused problems in the family?” at age 15 with the response categories “yes” or “no”.

*Loss of parent, parental separation or divorce* was measured by the question “Were your parents ever separated or divorced?” at age 15 and 18 as well as by the question “Have you lost any of your parents because they died?” at age 15 with the response categories “yes” and “no”.

*Emotional neglect* was measured by the following two questions regarding mother and father: “he/she seems emotionally attached to me” and “he/she understands my worries and problems” at age 15. Both questions were subject to the same response categories “Highly agree”, “agree”, “agree a little” and “do not agree at all” and was dichotomized to exposed (do not agree at all) and unexposed (highly agree/ agree/agree a little).

*Bullying* was measured by the question “Have you been bullied at school the last 6 months?” at age 15 and by the two questions “Have you been bullied in an unpleasant way at school during the last 6 months?” and “Have you been bullied in an unpleasant way at work during the last 6 months?” at age 18. All questions were subject to the same response categories “Never”, “once or twice”, “a few times”, “once a week” and “several times a week” and was dichotomized into exposed (once or twice/a few times/once a week/several times a week) and unexposed (never).

*Witness to violence* was measured by the question “Have you witnessed a very violent event?” at age 15 with the response categories “yes” or “no”.

At age 18, respondents had the opportunity to write an optional answer to the question “Did any other serious/negative life events happen during the last year?”. All answers were read by two of the authors separately, then compared and finally categorized according to the ACE-categories if the respondent was not already categorized as exposed.

Hereby, the whole childhood (up to age 18) is covered by the questions, however asking about different time periods dependent on the content of the question. Thus, the possible number of exposures to ACEs ranged from 0 (unexposed) to 6 (exposed to all categories) and finally, the respondents were categorized into one of the following four categories: having experienced 0, 1–2, 3 or > 4 ACEs.

### Confounder variables

Socioeconomic status was measured by information on equivalized disposable household income and highest attained educational level of a biological parent.

*Equivalized income* was obtained from Statistics Denmark [30] and calculated as a weighted average in DKK measured over a four-year period when the respondents were 7 to 10 years of age. It was categorized by the distribution of equivalized income in the source population into low (< 83.880 DKK.), medium (83.880–101.618 DKK.) and high (> 101.618 DKK.) income by the 33.3rd and 66.6th percentile.

*Highest educational level of the biological parent* was obtained from The Danish Educational Register [31] as the highest completed educational level of a biological parent, when the respondents were 13 years of age, and dichotomized into low (< 10 years) and high (> 10 years).

*Mental health* was measured by four questions at age 15 from the abbreviated version of the Epidemiological Studies Depression Scale for Children [32] asking about depressive symptoms over the past week with response categories ranging from 0 (not at all) to 3 (a lot). It was calculated as a sum-score from 0 to 12 using a cut-off point of 3 and above as recommended by Fendrich et al. [32] with higher values (< 2), indicating moderate mental health, and lower values (> 3), indicating a good mental health.

Information about g*ender* was obtained from the Danish Central Person Register [33].

### Health-behavioral variables

Weight and height were derived from questionnaires at age 21 and 28, and were used to calculate Body Mass Index (BMI, weight in kilograms divided by height in meters squared), which was dichotomized into normal weight (BMI < 25) and overweight (BMI > 25) by using guidelines from WHO [34]. Respondents who were more than 3 months pregnant were excluded from this study.

*Smoking* status at age 21 and 28 was measured by the question “Do you smoke?” with the response categories “daily”, “not every day”, “not every week”, “former smoker”, “never”. It was dichotomized into smoker (daily/not every day/not every week) and non-smoker (former smoker/never).

Information on *alcohol* at age 21 was measured by the question “how many days in the last month have you been drinking 5 units of alcohol in a row” and were dichotomized according to the recommendations from The Danish Health Authority’s guidelines [35] as high (> 3 days) or low (< 2 days). Information on alcohol at age 28 was measured by the question “how many units of alcohol do you usually drink during a week?” and were dichotomized according to the recommendations of The Danish Health Authority’s guidelines [35] as high (> 14 units for men and > 7 units for women) and low (< 14 units for men and < 7 units for women).

*Physical activity* at age 21 and 28 was measured by the question “How many hours a week during leisure time do you usually exercise or play sports where you are out of breath or sweating?” and was dichotomized according to the recommendations the guidelines of The Danish Health Authority’s [36] into high (> 4 h a week) and low (< 3 h a week).

### Statistical methods

Initially, characteristics of the entire study population collectively and categorized into > 1 ACEs and 0 ACEs were presented in Table 2 including the distribution of confounders and health-behavioral variables.

**Table 2:** Distribution (percentage) of variables included in the analysis for the complete study population (*n* = 2.255) on exposure, outcome, confounding- and health-behavioral variables and by experiencing > 1 ACEs compared to 0 ACEs

	All ***n*** = 2.255 (100)	**>** 1 ACEs ***n*** = 1.270 (56.3)	0 ACEs ***n*** = 985 (43.7)
**Exposure**			
Abuse	2.6		
Alcohol and/or drug abuser in the household	5.9		
Loss of parent, parental separation or divorce	23.5		
Emotional neglect	17.1		
Bullying	31.6		
Witness to violence	8.3		
**Categorized**			
0 ACEs	43.7		
1–2 ACEs	48.4		
3 ACEs	5.4		
> 4 ACEs	2.5		
**Outcome**			
**SRH, age 21**	***n*** **= 1.829**	***n*** **= 1.024**	***n*** **= 805**
Good	74.3	71.0	78.5
Moderate	25.7	29.0	21.5
**SRH, age 28**	***n*** **= 1.625**	***n*** **= 904**	***n*** **= 721**
Good	66.2	60.3	73.5
Moderate	33.8	39.7	26.5
**Confounding variables**			
**Equivalized income**	***n*** **= 2.226**	***n*** **= 1.249**	***n*** **= 977**
High	34.0	31.0	37.9
Medium	34.5	33.8	35.3
Low	31.5	35.2	26.8
**Highest educational level of the biological parent**	***n*** **= 2.207**	***n*** **= 1.227**	***n*** **= 980**
High	88.8	85.5	92.9
Low	11.2	14.5	7.1
**Mental health**	***n*** **= 2.226**	***n*** **= 1.253**	***n*** **= 973**
Good	66.1	57.4	77.4
moderate	33.9	42.6	22.6
**Gender (female)**	55.0	57.0	53.0
**Health-behavioral variables**			
**BMI, age 21**	***n*** **= 1.762**	***n*** **= 980**	***n*** **= 782**
Normal	73.7	70.3	78.0
Overweight	26.3	29.7	22.0
**BMI, age 28**	***n*** **= 1.652**	***n*** **= 923**	***n*** **= 729**
Normal	60.3	57.1	64.3
Overweight	39.7	42.9	35.7
**Smoking, age 21**	***n*** **= 1.742**	***n*** **= 966**	***n*** **= 776**
Non-smoker	71.7	58.5	75.6
smoker	28.3	31.5	24.4
**Smoking, age 28**	***n*** **= 1.641**	***n*** **= 915**	***n*** **= 726**
Non-smoker	78.2	76.1	81.0
Smoker	21.8	23.9	19.0
**Alcohol, age 21**	***n*** **= 1.744**	***n*** **= 969**	***n*** **= 775**
High	59.0	60.5	57.2
Low	41.0	39.5	42.8
**Alcohol, age 28**	***n*** **= 1.649**	***n*** **= 919**	***n*** **= 730**
High	94.4	94.6	94.2
Low	5.6	5.4	5.8
**Physical activity, age 21**	***n*** **= 1.741**	***n*** **= 965**	***n*** **= 776**
High	42.4	36.9	49.4
Low	57.6	63.1	50.6
**Physical activity, age 28**	***n*** **= 1.560**	***n*** **= 863**	***n*** **= 697**
High	33.9	32.7	35.4
Low	66.1	67.3	64.6

Logistic regression models were used to calculate the association between the four ACE-categories and SRH separately at age 21 and 28, using 0 ACEs as the reference category. Afterwards, a two-step adjustment model was applied controlling for confounding variables and finally health-behavioral variables. All estimations are presented as odds ratios (OR) and adjusted odds ratios (AOR) with 95% confidence intervals (95% CI).

Both confounding and health-behavioral variables were examined for correlations, using Spearman’s Rank correlation coefficient to minimize the risk of over-adjustment in the analysis - no value above *r* = 0.3 was found. Furthermore, all confounders were tested for interaction using Wald’s test with no significant interactions found.

Model 1 estimated the crude association between ACEs and SRH at age 21 and 28 (Model 1A and 1B) and in Model 2, the estimates were adjusted for the confounding variables equivalized income, highest education, mental health and gender at age 21 and 28, respectively (Model 2A and 2B). Additional adjustments (Model 3A and 3B) were calculated using the health-behavioral variables BMI, smoking, alcohol consumption and physical activity in order to analyze if the association between ACEs and SRH were further attenuated.

Finally, sensitivity analyses were made to investigate if the association between ACEs and SRH were dependent on either the categorization of ACEs or the cut-point in SRH. This was done by changing the ACEs categorization to 0–1, 2–3 and > 4 ACEs and the SRH dichotomization were changed to good (excellent/very good/good) and moderate (less good/poor).

All analyses were carried out using the statistical package STATA software version 16.1 (StataCorp LLC).

### Ethics

Use of the data was carried out under the same conditions and with the same purpose as originally collected. The study was approved by the Danish Data protection Agency. According to Danish Law, approval by the Ethics Committee and written consent were not required for questionnaire-based projects and usage of register-based data (The Act on Processing of Personal Data – Act No. 429 of 31 May 2000). The general ethical principles of Helsinki Declaration were conformed.

## Results

Table 2 shows a higher proportion (percentage) of participants having experienced at least one ACE compared to participants having experienced none (56.3 vs. 43.7). The most prevalent ACEs among the participants were “bullying” and “loss of parent, parental separation or divorce” with the least prevalent ACEs being “abuse” and “alcohol and/or drug abuser in the household”.

Comparing participants who had experienced at least one ACE with participants who had experienced none on confounding variables, ACEs were more common in the lower equivalized income group, among those with moderate mental health and those having low educated parents. When comparing participants on health-behavioral variables, ACEs were more common among participants who smoked, had a high BMI or low physical activity at age 21 and 28. Conversely, high alcohol consumption was more common among participants with 0 ACEs at age 21 and 28.

### The association between ACEs and SRH at age 21

Table 3, Model 1A, shows significantly higher crude odds of reporting moderate SRH at age 21 for all ACE exposure groups compared to the 0 ACEs reference group. The highest odds were seen in the > 4 ACEs group with 3.5-fold increased odds of reporting moderate SRH.

**Table 3:** Unadjusted and adjusted estimates of the association between ACEs and SRH at age 21, described by odds ratios with 95% confidence intervals

	Model 1A *n* = 1.829	Model 2A *n* = 1.775	Model 3A *n* = 1.626
0 ACEs	1	1	1
1–2 ACEs	1.4 (1.1; 1.8)	1.3 (1.0; 1.6)	1.0 (0.8; 1.3)
3 ACEs	1.7 (1.1; 2.8)	1.4 (0.9; 2.4)	1.3 (0.7; 2.2)
> 4 ACEs	3.5 (1.9; 6.5)	2.6 (1.3; 5.1)	2.2 (1.0; 4.7)

Model 1A = unadjusted association between ACEs and SRH at age 21

Model 2A = adjusted for equivalized income, highest educational level of the biological parent, mental health and gender

Model 3A = adjusted for Model 2A + BMI, smoking, alcohol and physical activity at age 21

All estimates attenuated when adjusted for confounding variables. In model 2A, however, those having experienced > 4 ACEs still had a 2.6-fold significantly higher risk of reporting moderate SRH at age 21. Adjusting for health-behavioral variables in Model 3A attenuated the association further, however, those reporting > 4 ACEs still had a 2.2-fold higher risk of reporting moderate SRH at age 21. Furthermore, significant exposure-response relationships between the cumulative numbers of ACEs and odds of moderate SRH were found in Model 1A (*p* < 0.001), 2A (*p* < 0.01) and 3A (*p* < 0.03).

### The association between ACEs and SRH at age 28

Table 4, Model 1B, reveals significantly higher crude odds of reporting moderate SRH in all ACE exposure groups compared to the 0 ACEs reference group. The highest odds of reporting moderate SRH was seen in the > 4 ACEs group, showing 3.7-fold increased odds of moderate SRH at age 28.

**Table 4:** Unadjusted and adjusted estimates of the association between ACEs and SRH at age 28, described by odds ratios with 95% confidence intervals

	Model 1B *n* = 1.625	Model 2B *n* = 1.552	Model 3B *n* = 1.415
0 ACEs	1	1	1
1–2 ACEs	1.7 (1.4; 2,1)	1.6 (1.3; 2.0)	1.4 (1.1; 1.8)
3 ACEs	2.6 (1.7; 3,9)	2.5 (1.6; 4.0)	1.8 (1.0; 3.0)
> 4 ACEs	3.7 (2.0; 6,8)	2.7 (1.4; 5.4)	2.4 (1.2; 4.9)

Model 1B = unadjusted association between ACEs and SRH at age 28

Model 2B = adjusted for equivalized income, highest educational level of the biological parent, mental health and gender

Model 3B = adjusted for Model 2B + BMI, smoking, alcohol and physical activity at age 28

All estimates attenuated when adjusted for confounding variables. In Model 2B, however, the > 4 ACEs group was still having a 2.7-fold significantly increased risk of reporting moderate SRH at age 28. Adjusting for health-behavioral variables in Model 3B further resulted in minor attenuations of all estimates, including the > 4 ACEs group, which now showed a 2.4-fold increased risk of reporting moderate SRH at age 28 compared to the 0 ACEs reference group. An exposure-response relationship between the cumulative numbers of ACEs and odds of moderate SRH were found in Model 1B (*p* < 0.001) and 2B (*p* < 0.01), but only to a lesser degree in model 3B (*p* < 0.08).

## Discussion

The aim of this study was to investigate the association between ACEs and SRH in a young Danish population and to examine if this association was affected when further adjusting for health-behavioral factors. Findings from this study add to the body of evidence showing an association between ACEs and SRH. Results showed increasing odds between ACE exposure groups and reporting moderate SRH at age 21 and 28. At both age points a more than 2.5-fold increased odds of moderate SRH was seen in those reporting > 4 ACEs compared to those reporting 0 ACEs when adjusted for potential confounders. At the same time, significant exposure-response relationships were found between the number of ACEs and increased odds of moderate SRH. When adjusting for the health-behavioral factors BMI, smoking, alcohol and physical activity, associations between ACEs and SRH at age 21 and 28 were further attenuated, however, still showing up to 2.4-fold increased odds of moderate SRH at age 28.

The adjusted odds ratios for participants with > 4 ACEs are comparable with previous findings in studies of both upper-middleclass Americans (AOR 2.2–95% CI 1.8; 2.7) [4], urban ethnic minorities Americans (AOR 1.75–95% CI 1.8; 2.7) [37] and British elderly (AOR 2.15–95% CI 1.62; 2.87) [22]. This means that our study, together with previous findings, indicates a consistent association between ACEs and SRH independent of the social security and welfare system and that the association is maintained across cultures, socioeconomic status and age groups. Furthermore, it seems that the negative effect of ACEs on SRH is observable already in young adulthood.

The adjusted analyses confirmed, to some extent, the confounding effect of the chosen variables on the association between ACEs and SRH, but they did not waive the possibility of residual confounding. Other potential confounders could include parental lifestyle during the child’s upbringing and childhood neighborhood.

Using mental health as a confounding variable could affect the analyses in Model 2A and 2B with the risk of adjusting with a potential mediator. But since mental health is collected at the same point in time as ACEs (age 15), we used it is a confounding variable due to its own relationship with SRH and based on its distribution between exposed and unexposed participants.

All health-behavioral factors were more prevalent among participants who had experienced at least one ACE at age 21 and 28, compared to 0 ACES despite high alcohol consumption. These findings support the literature suggesting that individuals who are exposed to ACEs are more likely to be developing these disadvantageous health-behavioral lifestyles. For example, Kendall-Tackett [38] propose four pathways in which ACEs lead to worse health in adult life: 1. behavioral, 2. social, 3. cognitive and 4. emotional pathways. Danese et al. [10] and Pechtel et al. [39] examined how allostatic toxic stress from ACEs leads to changed development in the nervous, endocrine and immune systems resulting in hampered cognitive, social and emotional functioning. From a theoretical perspective it is possible that the estimated associations in this study could be caused by the adaptation of disadvantageous health-behaviors which leads to the participants rating their SRH as moderate. However, adjusting for health-behavioral variables only slightly reduced the estimates, which indicate that the included health-behavioral variables did not fully explain the relationship between ACEs and SRH. Therefore, including different and/or other variables regarding health-related behavior or well-being, e.g. own substance abuse or mental disorders, may probably further attenuate the estimated association.

When altering the categorization in both ACEs and SRH in the sensitivity analysis, the exposure-response associations were still maintained and therefore not dependent on the categorization.

### Strengths and limitations

A major strength of the study was the prospective design using data from four survey rounds from the West Jutland Cohort Study. Furthermore, the study used information on ACEs collected in early and late adolescent, which is a major strength limiting the possibility of both recall- and survival bias. To our knowledge, the study, is the first prospective study in Scandinavia investigating the association between ACEs and young adulthood SRH. Finally, adjusting for register-based information about equivalized income and highest educational level of the biological parent is also considered a major strength.

However, some potential limitations need to be considered when interpreting the results of the study. The ACE proxy variables were collected from the West Jutland Cohort Study and though many of the selected items were based on validated abbreviated scales, inspired from the ACE-IQ, the measured construct within each ACE-category was not completely identical with the WHO’s ACE-IQ.

Furthermore, it was only possible to cover 6 out of the 13 ACE-categories from the WHO’s ACE-IQ by use of questions from the West Jutland Cohort Study. As an example, the authors used the question “were you abused?” to collect information about both physical, emotional and contact sexual abuse, which potentially could have resulted in an underestimated prevalence of abuse, which in the analyses possibly could have resulted in increased uncertainty of the estimated ORs as well as misclassification. Additionally, some questions in the West Jutland Cohort Study did not cover the entire lifespan of the respondents but asked only about the last year. Again, this could potentially have led to an underestimation of the true prevalence.

Another potential limitation relates to the collection of data. Due to the harsh nature of the ACE related questions, data attained through self-reports could potentially be wrongly answered, or not answered at all by the participants in fear of possible reprisals. In either case, this would most likely have resulted in bias towards the null hypotheses. An analysis of excluded respondents showed that they had a higher number of ACEs and more often rated their SRH as moderate compared to the included participants. Furthermore, it showed that a higher proportion of the excluded respondents were from the lower equivalized income group and had lower educated parents. This potential selection bias may have lessened the true associations consequently, creating a possible bias towards the null hypothesis. All though, loss to follow-up is a common problem in prospective studies, it does not necessary introduce bias on relative estimates as shown in a previous study on the present cohort [40].

The final limitation relates to the outcome as well confounding and health-behavioral variables, being categorized or dichotomized, which could result in loss of information and thereby information bias.

## Conclusion

This study showed an association between ACEs and moderate SRH in young adulthood and that experiencing multiple ACEs increased the odds of reporting moderate SRH. When adjusting for both confounding and health-behavioral variables those reporting > 4 ACES at age 21 and 28 consistently showed a 2-fold increased ORs compared to those reporting 0 ACEs. This result indicates that people who are exposed to ACEs could potentially be at higher risk of future health problems due to SRHs ability to predict future morbidity and mortality.

Thereby, this study contributes to the existing literature investigating ACEs negative impact on health and adds to the growing body of evidence, that the long-lasting negative impact of adverse childhood experiences on health can already be manifested in young adulthood. These results accentuate the growing need for early prevention in homes with children at risk of being exposed to ACEs.

Future research should focus on including more ACEs from WHO’s ACE-IQ covering the complete childhood period, e.g. variables regarding parents’ lifestyle to further explain the association between ACEs and SRH and further evaluate the consequences of ACEs in a life course perspective.

## Data Availability

The datasets used and/or analyzed during the current study are available from the authors on reasonable request.

## Abbreviations

ACEs: Adverse Childhood Experiences
ACE-IQ: Adverse Childhood Experiences International Questionnaire
AOR: Adjusted Odds Ratio
BMI: Body Mass Index
CI: Confidence Interval
DKK: Danish krone
E.g.: Exempli gratia
OR: Odds Ratio
SRH: Self-rated Health
SES: Socioeconomic status
US: United States
WHO: World Health Organization
